# Supplementary material for: The interactome of intact mitochondria by cross-linking mass spectrometry provides evidence for coexisting respiratory supercomplexes
Source: Mol Cell Proteomics. 2017 Dec 8;17(2):216–32. doi: 10.1074/mcp.RA117.000470 (PMC5795388; doi:10.1074/mcp.RA117.000470)
Supplement: Supplemental Data [file supp_RA117.000470_133922_0_supp_23978_fzffwf.zip › spectra_annotation/mito_DR_spectra_annotation/371-1-18-1-4-1.pdf]

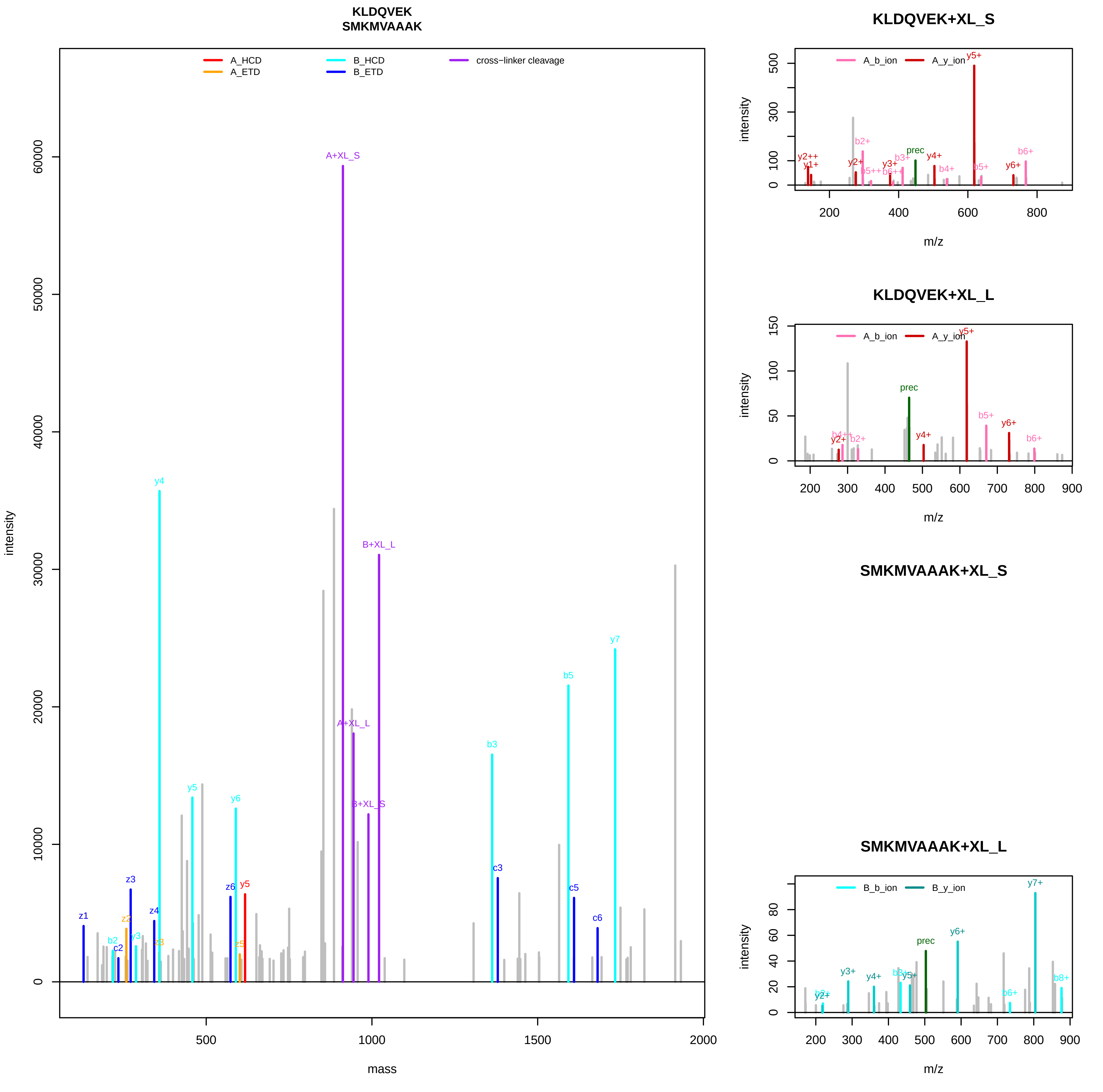
KLDQVEK
SMKMVAAAK
A_HCD
A_ETD
B_HCD
B_ETD
cross−linker cleavage
intensity
mass
A+XL_S
B+XL_L
A+XL_L
B+XL_S
y4
y5
y6
y7
b2
b3
b5
y3
z1
z2
z3
z4
z5
z6
c2
c3
c5
c6
y5
0
10000
20000
30000
40000
50000
60000
500
1000
1500
2000
KLDQVEK+XL_S
KLDQVEK+XL_L
SMKMVAAAK+XL_S
SMKMVAAAK+XL_L
A_b_ion
A_y_ion
B_b_ion
B_y_ion
prec
m/z
y1+
y2+
y2++
y3+
y4+
y5+
y6+
y7+
b2+
b3+
b4+
b4++
b5+
b5++
b6+
b6++
b8+
200
300
400
500
600
700
800
900
